# Fructo-oligosaccharides ameliorate steatohepatitis, visceral adiposity, and associated chronic inflammation via increased production of short-chain fatty acids in a mouse model of non-alcoholic steatohepatitis

**DOI:** 10.1186/s12876-020-01194-2

**Published:** 2020-02-27

**Authors:** Atsuko Takai, Kentaro Kikuchi, Mayuko Ichimura, Koichi Tsuneyama, Yuki Moritoki, Kotaro Matsumoto, Hiromichi Tsunashima, Takeshi Onda, Noriyuki Kuniyoshi, Tomoyuki Nariyama, Sho Ohyatsu, Juri Kubota, Kozue Nagumo, Shinpei Sato, Masumi Hara, Hiroshi Miyakawa

**Affiliations:** 1 Fourth Department of Internal Medicine, Teikyo University Mizonokuchi Hospital, 5-1-1 Futako, Takatsu-ku, Kawasaki-shi, Kanagawa 213-8507 Japan; 2 Department of Pathology and Laboratory Medicine, Institute of Biomedical Sciences, Tokushima University Graduate School, Tokushima-shi, Tokushima, Japan; 3 Department of General Internal Medicine and Clinical Laboratory Medicine, Akita University Graduate School of Medicine, Akita-shi, Akita, Japan; 4 Department of Gastroenterology, Teikyo University Mizonokuchi Hospital, Kawasaki-shi, Kanagawa Japan; 5 Department of Gastroenterology, Nippon Medical School Chiba Hokusoh Hospital, Inzai-shi, Chiba, Japan; 6 Division of Gastroenterology and Hepatology, Department of Medicine, Nihon University School of Medicine, Itabashi-ku, Tokyo, Japan

**Keywords:** Fructo-oligosaccharides, Monosodium glutamate, Non-alcoholic fatty liver disease, Non-alcoholic steatohepatitis, Short-chain fatty acids

## Abstract

**Background:**

Non-alcoholic fatty liver disease (NAFLD) is a hepatic manifestation of metabolic syndrome. Within the spectrum of NAFLD, non-alcoholic steatohepatitis (NASH) in combination with hepatic inflammation and fibrosis can lead to liver cirrhosis and hepatocellular carcinoma. Dysbiosis was reported to contribute to NASH pathogenesis. This study aimed to determine the effects of fructo-oligosaccharides (FOS) on steatohepatitis and visceral adiposity in an obese mouse model of NASH.

**Methods:**

Twelve newborn C57BL/6 J male mice were subcutaneously injected with monosodium glutamate (MSG) to induce obesity on a conventional diet. Six mice were also administered 5% FOS via drinking water from 10 weeks of age. At 18 weeks, histological characteristics of the liver and epididymal fat were compared between the groups. Hepatic mRNA expression of lipid metabolism enzymes and SCFA in feces and sera were measured.

**Results:**

Hepatic steatosis, inflammatory cell infiltration, and hepatocyte ballooning in the liver and increased hepatic mRNA expression of fatty acid synthase and glycerol-3-phosphate acyltransferase were observed in the MSG-treated mice. FOS treatment improved the liver pathology and blunted the increases in the mRNA expression levels of lipid metabolism enzymes. In addition, FOS inhibited adipocyte enlargement and formation of crown-like structures and reduced the M1 macrophage frequency in the epididymal fat of the MSG mice (39.4% ± 3.0% vs. 22.8% ± 0.7%; *P* = 0.001). FOS increased not only the fecal concentrations of n-butyric acid (0.04 ± 0.01 vs. 0.38 ± 0.14 mg/g, *P* = 0.02), propionic acid (0.09 ± 0.03 vs. 0.42 ± 0.16 mg/g, *P* = 0.02), and acetic acid (0.65 ± 0.16 vs. 1.48 ± 0.29 mg/g, *P* = 0.03) but also the serum concentration of propionic acid (3.9 ± 0.5 vs. 8.2 ± 0.5 μmol/L, *P* = 0.001).

**Conclusions:**

FOS ameliorates steatohepatitis, visceral adiposity, and chronic inflammation by increasing SCFA production.

## Background

Non-alcoholic fatty liver disease (NAFLD) is often accompanied by obesity, type 2 diabetes mellitus, and dyslipidemia and is therefore considered a hepatic manifestation of metabolic syndrome [1]. Among the various forms of NAFLD, non-alcoholic steatohepatitis (NASH) involves hepatic inflammation and fibrosis, which can lead to cirrhosis, and the reported five-year cumulative incidence of liver cancer among patients with cirrhosis is 20% in Japan [2].

Recent analyses revealed that bacterial flora and dietary microbial metabolites, such as SCFA, contributed to homeostasis as well as various diseases in humans [3]. An imbalance in the microbiota, the bacterial flora of the gastrointestinal tract, termed dysbiosis, was recently reported to be involved in inflammatory bowel disease, type 2 diabetes mellitus, and autism [4, 5].

In NASH, dysbiosis can stem from an unbalanced diet or obesity, and a reduction in the production of SCFA can lead to a dysfunctional intestinal mucosal barrier and immunological disorders [6]. Studies showed that a large number of pathogen-associated molecular patterns could access the liver through disrupted intestinal mucosal epithelia, causing Kupffer cell hypersensitivity, and could lead to steatohepatitis [7–9].

We recently reported that fructo-oligosaccharides (FOS), which are prebiotics, ameliorated dysbiosis and improved intestinal barrier function, thereby suppressing Kupffer cell activation and improving steatohepatitis in mice fed a methionine/choline-deficient diet (MCD) [10]. FOS, which are found in foods that are not digested or absorbed in the upper gastrointestinal tract, are fermented selectively by beneficial intestinal bacteria. Therefore, FOS induce favorable changes in the intestinal bacterial flora and have health-promoting effects in the entire body [11, 12].

MCD-fed mice in the NASH model do not exhibit obesity or insulin resistance; thus, the mechanism underlying the effect of FOS on lipid metabolism in the liver or visceral fat levels cannot be examined, whereas the monosodium glutamate (MSG)-treated mouse is an appropriate animal model of NASH. Neonatal administration of MSG in rodents was previously demonstrated to affect the arcuate nucleus and the ventromedial nucleus of hypothalamus and to impair leptin signaling, resulting in obesity in rodents that were fed a conventional diet [13, 14]. Nakanishi et al. first reported that an injection of MSG in Institute of Cancer Research mice leads to the development of a significant human NASH-like liver histology [15]. In recent years, MSG-treated mice have been widely used in human NASH research [16–19]. Therefore, we investigated the mechanism by which FOS ameliorate steatohepatitis and tested our hypothesis that FOS alleviate visceral adiposity using an MSG-treated obese mouse model of NASH.

## Methods

### Generation of the NASH mouse model and FOS administration

The MSG-treated mouse model of NASH was described previously [20, 21]. Briefly, MSG (Wako Pure Chemical Industries, Osaka, Japan) in normal saline at a dose of 4 mg/g body weight was subcutaneously injected into the backs of 12 male C57BL/6 J mice (Sankyo Labo Service, Tokyo, Japan) at 1 week of age. All mice were fed a conventional diet and purified water and housed under conventional conditions. Six of the MSG-treated mice were administered 5% FOS (Meioligo W; Meiji Corporation, Tokyo, Japan) in their drinking water in addition to the conventional diet, starting at 10 weeks of age. These mice in the MSG + FOS group were housed under the same conditions as those in the MSG group. The control group comprised six male mice aged 1 week that were subcutaneously injected with saline at the same volume as MSG and fed the same diet and purified water and housed under the same conditions as the mice in the MSG group. The body weight of all mice was measured periodically up to 18 weeks of age.

This study was approved by the Teikyo University School of Medicine Animal Ethics Committee (approval number: 14–030) and was conducted in accordance with the relevant institutional guidelines.

### Serological and histological analyses

At 18 weeks of age, the mice were anesthetized with intraperitoneal administration of 75 mg/kg ketamine hydrochloride and 1 mg/kg medetomidine chloride, and the levels of blood glucose, insulin, total cholesterol, adiponectin and alanine aminotransferase (ALT) in serum samples of venous blood were measured. The serum concentrations of n-butyric acid, propionic acid, and acetic acid were measured using the GCMS-QP2010 Ultra/SE gas chromatography system (Shimadzu Corporation, Kyoto, Japan) [22]. The mice were sacrificed by cervical dislocation, and the livers were weighed and perfused with PBS containing 0.5% bovine serum albumin and 0.04% EDTA (PBS buffer) that was administered from the inferior vena cava before their removal. Half of each liver was fixed in 4% paraformaldehyde, stained with hematoxylin and eosin, and histopathologically evaluated according to the NAFLD activity score (NAS) [23]. The remainder of each liver was snap-frozen in liquid nitrogen and stored at − 80 °C until analysis by real-time PCR.

The epididymal fat of the mice was excised from the peritoneal cavity and weighed. Half of the epididymal fat was fixed in 4% paraformaldehyde, stained with hematoxylin and eosin, and evaluated histopathologically. The remaining fat was sliced thinly before dissociation by passing through a cell strainer with a mesh size of 40 μm (BD Falcon, Durham, NC, USA). The dissociated cells were suspended in PBS buffer and centrifuged for 5 min at 1000 rpm. The supernatant was discarded, and the pellet was resuspended in PBS buffer, layered on Lymphoprep (Axis-Shield Proc. AS, Oslo, Norway) at a relative density of 1.077, and centrifuged for 15 min at 1400 rpm to isolate the stromal vascular fraction (SVF), which was collected using a pipette and washed in PBS buffer. The number of viable cells in the SVF was counted using trypan blue staining.

### Analysis of the bacterial flora and SCFA in feces

Feces were collected from the cecum to analyze the bacterial flora and measure SCFA concentrations. The partial amino acid sequence of bacterial 16 s rDNA was analyzed using the terminal restriction fragment length polymorphism (T-RFLP) technique based on Nagashima’s method [24]. Briefly, 20 mg feces was dissolved in 0.2 ml distilled water and rinsed via centrifugation. The resultant pellet was dissolved in 250 μl Tris-EDTA buffer containing 100 mM Tris-HCl and 40 mM of EDTA. After centrifugation with 0.6 g DNA extraction beads, the supernatant was collected and mixed with 150 μl benzyl chloride and 50 μl 10% sodium lauryl sulfate for 30 min at 50 °C. After centrifugation with 150 μl 3 M sodium acetate, the supernatant was collected and mixed with isopropyl alcohol for DNA extraction. Next, 0.5 U of HotStarTaq DNA polymerase (Qiagen, Tokyo, Japan) was added to 10 ng DNA, and bacterial 16 s rDNA was amplified via PCR using the 5′ terminal fluorolabeled 516f and 1510r primer sets [24]. After the digestion of DNA with the restriction enzyme Bsl I, the resultant fragments were analyzed using an ABI PRISM 3130xl DNA sequencer (Applied Biosystems, Carlsbad, CA, USA) and the GeneMapper software (Applied Biosystems). The length of each fragment was determined based on operational taxonomic units, and peak area ratios were presented as percentages.

In addition, 1 g feces was pulverized under sterile conditions. The fecal SCFA concentrations were analyzed using the Prominence HPLC system (Shimadzu Corporation, Kyoto, Japan).

### Gene expression analysis

Total RNA was extracted from the liver using RNAiso Plus (Takara Bio, Shiga, Japan), and cDNA was synthesized using the ReverTra Ace qPCR RT master mix (Toyobo, Osaka, Japan). Real-time PCR was performed with the Thunderbird SYBR qPCR mix (Toyobo) using the LightCycler® Nano system (F. Hoffmann-La Roche, Basel, Switzerland), as described previously [25]. Specific primers were designed using the Primer-BLAST primer designing tool (National Center for Biotechnology Information, Bethesda, MD, USA) and were synthesized by FASMAC (Kanagawa, Japan). The relative mRNA expression levels were normalized to the expression level of β-actin.

### Flow cytometric analysis

Flow cytometry was used to analyze M1 and M2 adipose tissue macrophages (ATM). All antibodies and the cell-staining buffer were obtained from BioLegend (San Diego, CA, USA). To prevent non-specific staining, a total of 1 × 10^6^ SVF cells were incubated with 1 μl purified anti-mouse CD16/32 antibody (clone 93) in 24 μl cell-staining buffer containing sodium azide for 10 min at 4 °C. Fluorescein isothiocyanate-conjugated anti-mouse F4/80 antibody (clone BM8), phycoerythrin (PE)-conjugated anti-mouse CD11c antibody (clone N418), allophycocyanin (APC)-conjugated anti-mouse CD11b antibody (clone M1/70), APC/Cy7-conjugated anti-mouse CD86 antibody (clone GL-1), PE-conjugated American Hamster IgG isotype control antibody (clone HTK888) and APC/Cy7-conjugated Rat IgG2a-kappa isotype control antibody (clone KLH) in 25 μl cell-staining buffer were added to the cells and incubated in the dark for 10 min at 4 °C After washes, the labeled cells were resuspended in 200 μl PBS buffer, transferred to the wells of 96-well round-bottomed plates, and analyzed on a BD FACSArray flow cytometer (BD Immunocytometry Systems, San Jose, CA, USA).

### Statistical analysis

Mouse body weight, epididymal fat weight, total serum cholesterol level, serum ALT level, serum and fecal levels of SCFA, NAS, mRNA expression levels, and frequencies of M1 and M2 ATM were expressed as means ± standard error of the mean. The mean fluorescence intensity (MFI) ratios of CD86 was calculated as the sample MFI divided by the isotype control MFI. All statistical analyses were performed using one-way ANOVA with Tukey’s post hoc test using GraphPad Prism version 6.0 for Macintosh (GraphPad Software, San Diego, CA, USA), and differences were considered significant at a *P* value of < 0.05.

## Results

### Physical and serological characteristics

**Fig. 1:**
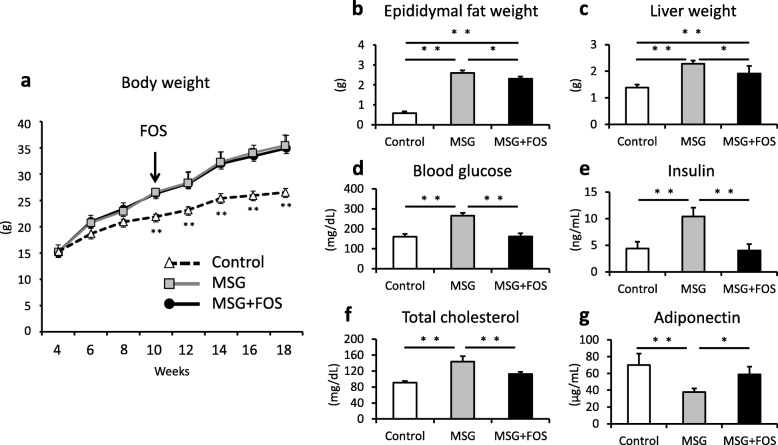
Physical and serological findings of the control, MSG, and MSG + FOS mice. **a** Changes in mean body weight, and at 18 weeks of (**b**) epididymal fat weight, **c** liver weight, **d** serum blood glucose levels, **e** serum insulin levels, **f** serum total cholesterol levels, and (**g**) serum adiponectin levels. *p < 0.05, **p < 0.01. FOS, fructo-oligosaccharides; MSG, monosodium glutamate

At 10 weeks of age, the mice in both the MSG and MSG + FOS groups were heavier than the control mice (MSG, 26.5 ± 0.6 g; MSG + FOS, 26.3 ± 0.5 g; control, 23.1 ± 0.5 g; *p* = 0.001 for both; Fig. 1a). The mean body weights did not differ between the MSG and the MSG + FOS mice at 18 weeks of age (34.9 ± 2.4 and 35.6 ± 2.0 g, respectively; *p* = 0.9). Conversely, at 18 weeks of age, the MSG + FOS mice exhibited a lower mean epididymal fat weight than the MSG mice (MSG, 2.7 ± 0.2 g; MSG + FOS, 2.3 ± 0.1 g; *p* = 0.04; Fig. 1b). Moreover, the MSG + FOS mice exhibited a lower mean liver weight than the MSG mice (MSG, 2.3 ± 0.1 g; MSG + FOS, 1.9 ± 0.3 g; *p* = 0.04; Fig. 1c).

The mean blood glucose and insulin levels of the MSG mice (265.4 ± 14.1 mg/dL, 10.4 ± 1.7 ng/dL) were higher than that of the control mice (160.0 ± 14.0 mg/dL, 4.4 ± 1.3 ng/dL; *p* = 0.001 for both). Compare to the MSG mice, MSG + FOS mice had lower levels of blood glucose and insulin (161.2 ± 15.8 mg/dL, 4.0 ± 1.2 ng/dL; *p* = 0.001 for both; Fig. 1d, e). The mean total cholesterol level of the MSG mice (148.6 ± 14.5 mg/dL) was higher than that of the control mice (85.6 ± 2.2 mg/dL; *p* = 0.001) and MSG + FOS mice (115.6 ± 8.7 mg/dL; *p* = 0.003; Fig. 1f). The mean adiponectin level of the MSG mice (37.7 ± 4.4 μg/mL) was lower than that of control mice (69.8 ± 13.8 μg/mL; *p* = 0.003; Fig. 1g). In MSG + FOS mice, serum adiponectin level (58.7 ± 9.4 μg/mL) was increased compare to MSG mice (*p* = 0.04).

### Histological characteristics of the liver

**Fig. 2:**
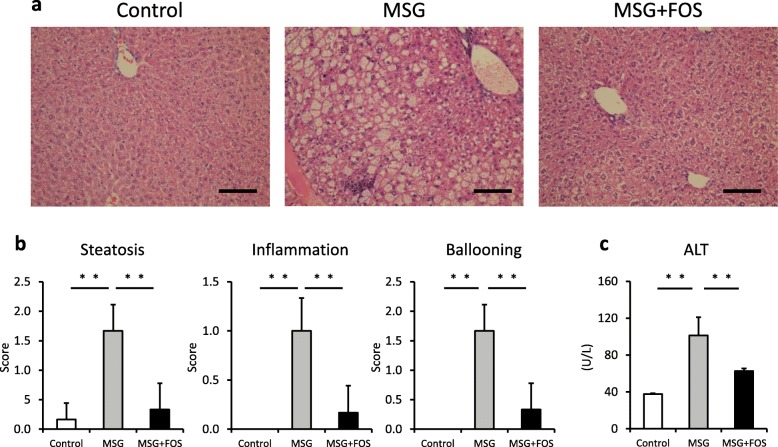
Liver findings and serum ALT levels in the control, MSG, and MSG + FOS mice at 18 weeks. **a** Histological findings (hematoxylin/eosin staining, 100× magnification, scale bar = 100 μm), **b** Mean NAFLD activity score, **c** Mean serum ALT levels. **p < 0.01. ALT, alanine aminotransferase; FOS, fructo-oligosaccharides; MSG, monosodium glutamate; NAFLD, non-alcoholic fatty liver disease

Hepatic steatosis, inflammatory cell infiltration, and ballooning hepatocytes were observed in the livers of the MSG mice (Fig. 2a), whereas these changes were less marked in the livers of the MSG + FOS mice. According to the NAS, steatosis (MSG, 1.7 ± 0.4; MSG + FOS, 0.3 ± 0.4; *p* = 0.001), lobular inflammation (MSG, 1.0 ± 0.3; MSG + FOS, 0.2 ± 0.1; *p* = 0.001) and ballooning degeneration (MSG, 1.7 ± 0.4; MSG + FOS, 0.3 ± 0.4; *p* = 0.001) were less severe in the MSG + FOS mice compared with the MSG mice (Fig. 2b).

The mean serum ALT level of the MSG mice was higher than that of the control mice (MSG, 108.3 ± 29.0 U/L; control, 39.2 ± 0.9 U/L; *p* = 0.001; Fig. 2c). Conversely, the mean serum ALT level of the MSG + FOS mice was lower than that of the MSG mice (MSG + FOS, 73.5 ± 7.0 U/L; *p* = 0.001).

### The hepatic mRNA expression levels of lipid biosynthesis enzymes

**Fig. 3:**
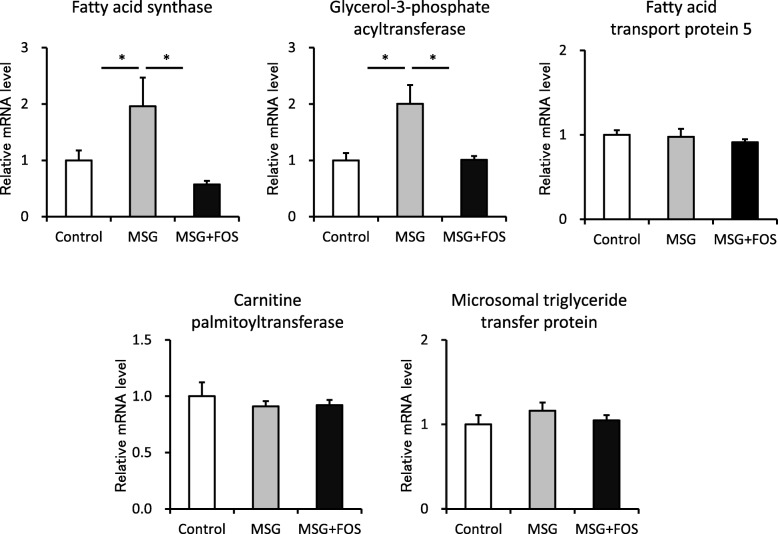
Hepatic lipid metabolism-related genes in the control, MSG, and MSG + FOS mice at 18 weeks. mRNA levels were determined by real-time PCR and are expressed relative to the levels in the control mice after normalization to β-actin mRNA expression. *p < 0.05, **p < 0.01. FOS, fructo-oligosaccharides; MSG, monosodium glutamate

In the MSG mice, the hepatic mRNA levels of enzymes linked to lipid biosynthesis were elevated. The mean relative mRNA expression levels of fatty acid synthase (control, 1.0 ± 0.17; MSG, 1.96 ± 0.51; *p* = 0.02) and glycerol-3-phosphate acyltransferase (control, 1.0 ± 0.13; MSG, 2.0 ± 0.33; *p* = 0.01) were significantly higher in the MSG mice than in the control mice (Fig. 3). Additionally, the hepatic mRNA expression levels of these enzymes were lower in the MSG + FOS mice (fatty acid synthase, 0.57 ± 0.06; *p* = 0.02; glycerol-3-phosphate acyltransferase, 1.01 ± 0.07; *p* = 0.01) than the MSG mice. There were no differences among groups in molecules involved in lipid uptake (fatty acid transport protein 5), lipolysis (carnitine palmitoyltransferase), or lipid transfer (microsomal triglyceride transfer protein).

### Histopathological characteristics of the epididymal fat and the frequency of M1-like ATM

**Fig. 4:**
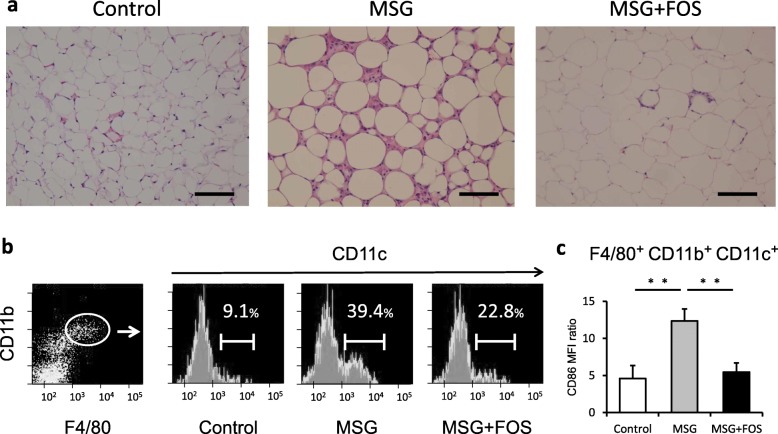
Visceral fat findings in the control, MSG, and MSG + FOS mice at 18 weeks. **a** Histological findings (hematoxylin/eosin staining, 100× magnification, scale bar = 100 μm), **b** Flow cytometric analysis of ATM. ATM were defined as F4/80^+^ CD11b^high^ cells. Among the ATM, M1-like macrophages were defined as CD11c^+^ cells. **c** MFI ratio of CD86 in F4/80^+^ CD11b^high^ CD11c^+^ cells. ATM, adipose tissue macrophages; FOS, fructo-oligosaccharides; MSG, monosodium glutamate; MFI, mean fluorescence intensity

Enlarged adipocytes and crown-like structures were observed in the epididymal fat of the MSG mice (Fig. 4a), whereas FOS treatment led to smaller adipocytes and prevented the formation of crown-like structures in the MSG + FOS mice. To assess the numbers of macrophages in the epididymal tissue, F4/80^+^ and CD11b^high^ cells were collected from the SVF, and M1-like ATM were identified as CD11c^+^ cells [26]. The proportion of M1-like ATM was 39.4% ± 3.0% of the SVF cells in the MSG mice, which was significantly higher than that in the control mice (9.1% ± 0.4%; *p* = 0.001; Fig. 4b). In the MSG + FOS mice, the proportion of M1-like ATM (22.8% ± 0.7%; *p* = 0.001) was significantly lower than that in the MSG mice. The MFI ratio of CD86 in M1-like ATM was 4.6 ± 1.8 in control mice, 12.4 ± 1.6 in MSG mice (vs. control mice; *p* = 0.001) and 5.5 ± 1.2 in MSG + FOS mice (vs MSG mice; *p* = 0.01; Fig. 4c).

### The bacterial composition of feces

The analysis of the bacterial composition of feces revealed that the frequency of *Clostridium* cluster XI, which was 3.6% in the control mice, was reduced to 1.3% in the MSG mice (*p* = 0.04), whereas the frequency of the bacteria classified as “others” was reduced from 17.5% in the control mice to 8.4% the MSG mice (*p* = 0.01). Conversely, the frequency of the genus *Prevotella*, which was 1.3% in the control mice, was increased to 8.7% in the MSG mice (*p* = 0.01) (Additional file 1: Figure S1). The feces of the MSG + FOS mice displayed a recovery of bacterial balance. Specifically, the frequencies of *Clostridium* cluster XI, “others”, and the genus *Prevotella* were 7.8, 12.1, and 1.8%, (*p* < 0.05 for both) respectively.

### The levels of SCFA in feces and serum

**Fig. 5:**
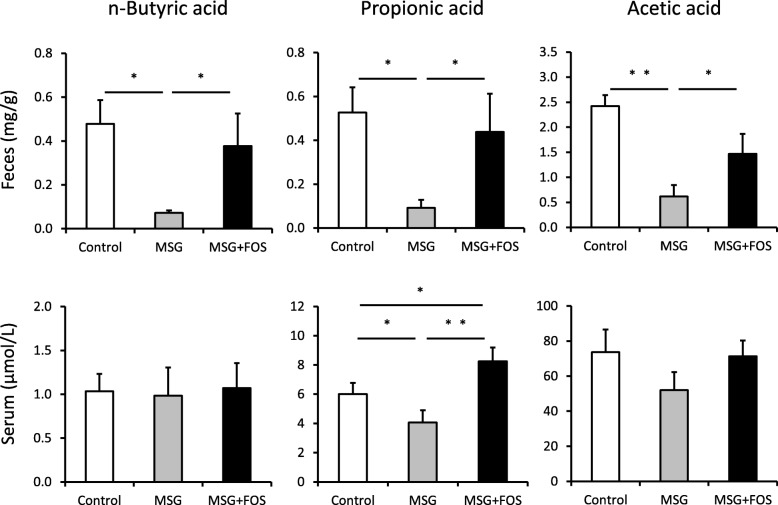
Fecal and serum SCFA concentrations in the control, MSG, and MSG + FOS mice at 18 weeks. *p < 0.05, **p < 0.01. FOS, fructo-oligosaccharides; MSG, monosodium glutamate; SCFA, short-chain fatty acids

The analysis of the SCFA levels in feces revealed that the mean fecal concentrations of n-butyric acid, propionic acid, and acetic acid were significantly lower in the MSG mice than the control mice (0.04 ± 0.01, 0.09 ± 0.03, and 0.65 ± 0.16 mg/g vs. 0.48 ± 0.11, 0.56 ± 0.09, and 2.46 ± 0.43 mg/g; *p* = 0.02, 0.02, and 0.007, respectively; Fig. 5). Conversely, the mean fecal concentrations of n-butyric acid, propionic acid, and acetic acid in the MSG + FOS mice (0.38 ± 0.14, 0.42 ± 0.16, and 1.48 ± 0.29 mg/g, respectively) were significantly higher than those in the MSG mice (*p* = 0.02, 0.02, and 0.03, respectively).

The mean serum concentrates of n-butyric acid and acetic acid did not differ among the three groups (*p* > 0.05 in all cases). However, the mean serum concentration of propionic acid was significantly lower in the MSG mice (3.9 ± 0.5 μmol/L) than in the control mice (5.9 ± 0.4 μmol/L; *p* = 0.03). In the MSG + FOS mice, the mean serum concentration of propionic acid (8.2 ± 0.5 μmol/L) was significantly higher than that in the MSG mice (*p* = 0.001).

## Discussion

The current study utilizing an obese mouse model of NASH revealed that FOS ameliorated dysbiosis and increased SCFA production by the intestinal bacterial flora. Hepatic steatosis and inflammatory cell infiltration were also ameliorated by the administration of FOS. In addition, visceral adiposity and associated chronic inflammation was significantly reduced by FOS.

In a previous study to elucidate the mechanism underlying the FOS-mediated amelioration of steatohepatitis, we reported that the increased SCFA production by the bacteria provided nutrients to intestinal epithelial cells, improved intestinal barrier function, increased immunoglobulin A production, and suppressed Kupffer cell activation in MCD-fed mice [10]. In the current study, we focused on the effect of SCFA on lipid biosynthesis in the liver and visceral adiposity and associated chronic inflammation using an obese mouse model of NASH.

There are three additional potential explanations for the observed beneficial effects of SCFA in the present study. First, SCFA promotes glucagon-like peptide-1 (GLP-1) secretion from the L cells of the intestinal tract [27], given that several recent clinical studies demonstrated that treatment with GLP-1, which regulates hepatic lipid accumulation [28]. Recent studies have shown the efficacy of GLP-1 in treating NAFLD [29, 30]. Second, adipocytes express SCFA receptor G-protein-coupled receptor 43 (GPR43). Kimura et al. reported that GPR43 can act to suppress insulin signaling in adipocytes and inhibit fat accumulation in adipose tissue as well as promote the metabolism of unincorporated lipids and glucose in the liver [31]. Third, SCFA can act as a ligand for peroxisome proliferator-activated receptor-γ (PPARγ), resulting in improved insulin sensitivity. den Besten et al. reported that SCFA-induced reduction of hepatic steatosis was absent in mice lacking hepatic PPARγ [32]. Mattace et al. reported that butyrate supplementation decreased pro-inflammatory markers interleukin-6 and nuclear factor-kappa B, thus increasing the threshold for inflammatory responses in the liver of high-fat diet-fed rats [33].

In the current study, we found that FOS significantly increased the serum concentration of propionic acid and reduced the mRNA expression levels of fatty acid synthase and glycerol-3-phosphate acyltransferase. Propionic acid was reported to reduce the hepatic mRNA and protein expression of lipid biosynthetic enzymes [34], increase glucose transporter type 4 (GLUT4) and improve insulin sensitivity [35], and inhibit lipopolysaccharide-stimulated tumor necrosis factor-α release by neutrophils [36]. Our future studies should elucidate the roles of SCFA and propionic acid in detail.

The effect of FOS on liver fibrosis was not evaluated, which was a limitation of the current study. MSG mice do not exhibit severe hepatic fibrosis, albeit displaying severe steatosis and inflammation compared to other NASH model animals [37]. The present study results suggested that the administration of FOS is an effective treatment for not only early-stage NASH but also visceral adiposity and associated chronic inflammation. Similar to MSG mice, ob/ob mice affect leptin signaling resulting obesity, hyperglycemia, and hepatic steatosis. However, MSG mice show more hepatic inflammation than ob/ob mouse [37].

Various methods to date were proposed to abrogate dysbiosis. In a study examining the outcomes following the administration of useful bacteria as probiotics in a NASH animal model, Velayudham et al. confirmed the attenuation of hepatic fibrosis and the downregulation of the hepatic mRNA expression levels of toll-like receptor 4 and CD14 in MCD mice that were fed VSL#3, is a probiotic formula including eight useful bacterial species, for 10 weeks. However, the authors could not confirm whether the treatment was associated with a significant alleviation of steatosis or inflammation [38]. Conversely, Endo et al. demonstrated that the tight junctions between the intestinal epithelial cells were reinforced and that steatosis and fibrosis were improved in the livers of choline-deficient/L-amino acid-defined diet-fed rats that were administered butyric acid-producing bacteria for 8–50 weeks [39]. However, abrogating dysbiosis by the administration of a single bacterial species may not be feasible. Recent clinical trials reported that synbiotic yogurt consumption improved hepatic steatosis in patients with NAFLD [40]. Therefore, investigating the effect of FOS on NASH remains an important approach.

## Conclusions

In conclusion, the current study provides a potential dietary strategy for the prevention and treatment of NASH, visceral adiposity, and associated chronic inflammation. At present, there are no approved therapies for NASH. Prebiotics such as FOS are found in onion, garlic, soybeans, and burdock and produced industrially as syrups. Thus, FOS are more appropriate for habitual consumption compared with probiotics. The effects of FOS on lifestyle-related diseases have been increasingly reported [41]. Future studies should elucidate the contribution of FOS to human health.

## Supplementary information

**Additional file 1:Figure S1.** Terminal restriction fragment length polymorphism analysis in the control, MSG, and MSG + FOS mice at 18 weeks. FOS, fructo-oligosaccharides; MSG, monosodium glutamate.

## Data Availability

The datasets used and/or analyzed during the current study are available from the corresponding author on reasonable request.

## Abbreviations

ALT: Alanine aminotransferase
ATM: Adipose tissue macrophages
FOS: Fructo-oligosaccharides
MCD: Methionine/choline-deficient diet
MSG: Monosodium glutamate
NAFLD: Non-alcoholic fatty liver disease
NASH: Non-alcoholic steatohepatitis
SCFA: Short-chain fatty acids
SVF: Stromal vascular fraction
