# Supplementary material for: Fructo-oligosaccharides ameliorate steatohepatitis, visceral adiposity, and associated chronic inflammation via increased production of short-chain fatty acids in a mouse model of non-alcoholic steatohepatitis
Source: BMC Gastroenterol. 2020 Feb 27;20:46. doi: 10.1186/s12876-020-01194-2 (PMC7045471; doi:10.1186/s12876-020-01194-2)
Supplement: Supplementary file 1 — Additional file 1:Figure S1. Terminal restriction fragment length polymorphism analysis in the control, MSG, and MSG + FOS mice at 18 weeks. FOS, fructo-oligosaccharides; MSG, monosodium glutamate. [file 12876_2020_1194_MOESM1_ESM.pptx]

## Slide 1
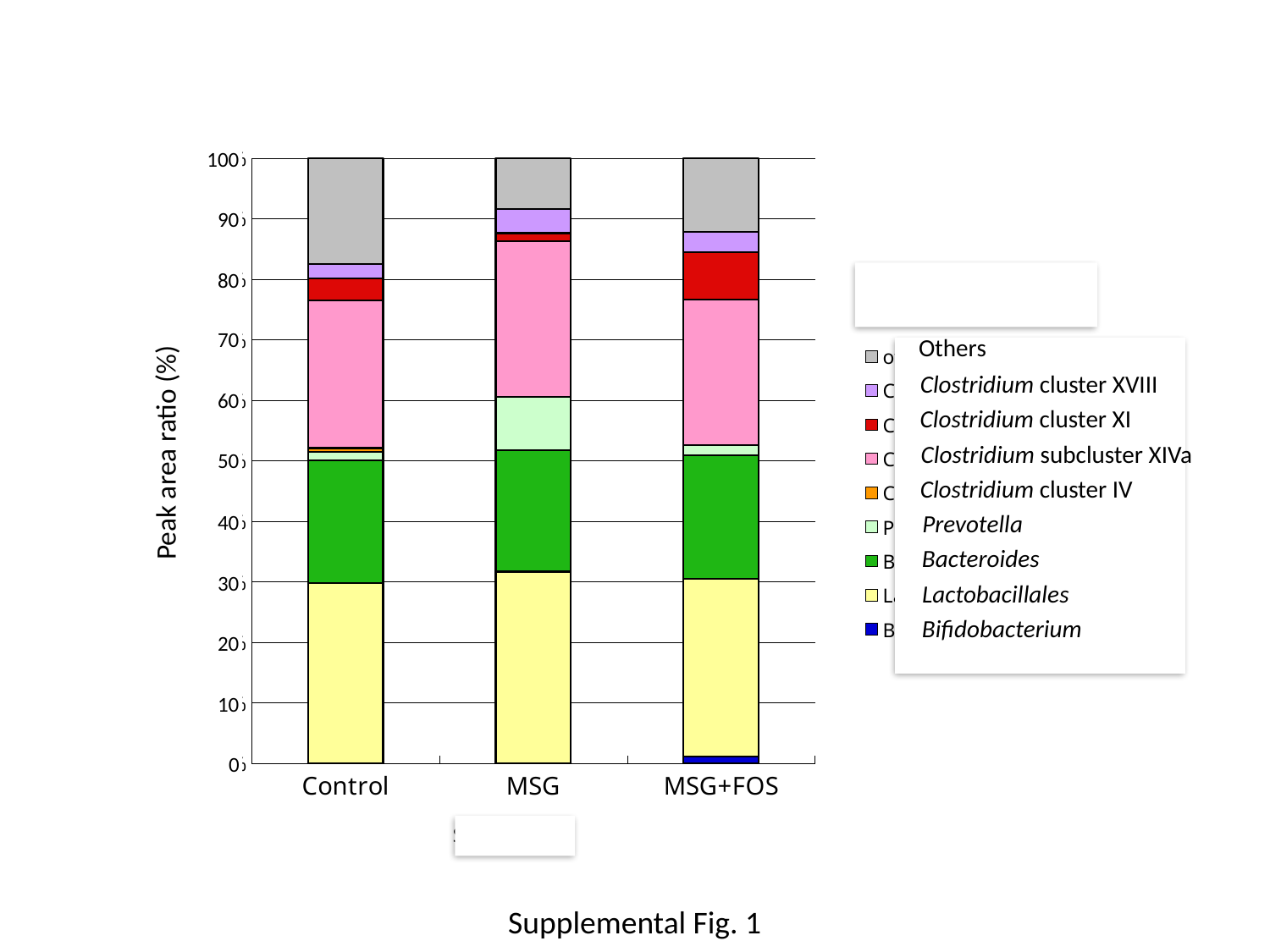

### Chart: 推定される分類群
| Category | Bifidobacterium | Lactobacillales　目 | Bacteroides | Prevotella | Clostridium cluster IV | Clostridium subcluster XIVa | Clostridium cluster XI | Clostridium cluster XVIII | others |
|---|---|---|---|---|---|---|---|---|---|
| Control | 0.0 | 29.82938862820867 | 20.31206872757384 | 1.28950455423682 | 0.684860612751863 | 24.42782224675685 | 3.56003312172233 | 2.405637593154843 | 17.49068451559481 |
| MSG | 0.0 | 31.68446684170408 | 20.09930549673922 | 8.728593207419328 | 0.0 | 25.85118997205048 | 1.270432794105192 | 3.952104683662235 | 8.413907004319471 |
| MSG+FOS | 1.194360221809756 | 29.3215665025766 | 20.34447377825941 | 1.78876194648436 | 0.0 | 24.03822874995677 | 7.764494299120368 | 3.425139207525852 | 12.12297529426684 |
100
90
80
70
Others
Clostridium cluster XVIII
60
Clostridium cluster XI
Peak area ratio (%)
Clostridium subcluster XIVa
50
Clostridium cluster IV
Prevotella
40
Bacteroides
30
Lactobacillales
Bifidobacterium
20
10
0
Supplemental Fig. 1
